# Cerebellar Transcranial Direct Current Stimulation Induces a Predominant Motor Rather than Perceptive Contribution to Emotion Facial Expression Processing

**DOI:** 10.3390/brainsci16080858

**Published:** 2026-08-13

**Authors:** Nicola Loi, Damiano Sottana, Mohammed Zeroual, Mattia Solinas, Matteo Spinelli, Francesca Ginatempo, Franca Deriu

**Affiliations:** 1 Department of Biomedical Sciences, University of Sassari, Viale San Pietro 43/b, 07100 Sassari, Italy; nloi@uniss.it (N.L.); mattiasolinas.02122002@gmail.com (M.S.); mspinelli@uniss.it (M.S.);; 2 Unit of Endocrinology, Nutritional and Metabolic Disorders, AOU Sassari, 07100 Sassari, Italy

**Keywords:** cerebellum, transcranial direct current stimulation, transcranial magnetic stimulation, cerebellar brain inhibition, event-related potentials, facial expressions

## Abstract

**Highlights:**

**What are the main findings?**

- Cerebellar cathodal tDCs selectively modulated the cerebello–thalamo–motor cortex pathway during passive viewing of emotional facial expressions, with no effects for neutral faces.
- Cerebellar neuromodulation did not alter early occipitotemporal components (P100 and N170 waves) or the recognition of emotional face expressions, suggesting its limited role in early perceptual processing.

**What are the implications of the main findings?**

- These findings support the hypothesis that cerebellar contributions to facial emotion processing are mediated predominantly by motor networks rather than early visual-perceptual mechanisms.
- The study provides new neurophysiological evidence for a functional dissociation between cerebellar motor and perceptual pathways during social-emotional processing, helping to clarify the cerebellum’s role in emotions’ recognition.

**Abstract:**

**Background**: The cerebellum has recently been proposed as a key contributor to facial expression (FE) processing, although the neural pathways underlying its involvement remain poorly understood. This study investigated whether cerebellar modulation of FE processing preferentially relies on motor or perceptual circuits. **Methods:** Sixteen healthy young participants underwent cerebellar cathodal transcranial direct current stimulation (tDCs) and sham stimulation in a randomized crossover design. Cerebellar–motor cortex interactions were assessed using cerebellar brain inhibition (CBI) measured by a paired-pulse transcranial magnetic stimulation protocol, while early perceptual processing was evaluated through P100 and N170 event-related potentials (ERPs). Behavioral performance was assessed using a task involving recognition of neutral, happy, and fearful faces. **Results:** Cerebellar tDCs selectively modulated the cerebello–thalamo–primary motor cortex pathway, producing a significant reduction in CBI during the passive viewing of emotional (happy and fearful) expressions while exerting no significant effects on occipitotemporal ERP components associated with early face perception or on behavioral measures of emotion recognition, including recognition accuracy and reaction times. **Conclusions:** These findings indicate that cerebellar contributions to facial emotion processing are predominantly mediated through motor rather than early perceptual pathways. The cerebellum appears to modulate motor network activity during emotional face observation without directly influencing occipitotemporal perceptual mechanisms, although this physiological modulation was not accompanied by measurable changes in behavioral performance. These findings support the hypothesis that cerebellar involvement in social cognition is primarily related to the optimization of emotion-related motor processing.

## 1. Introduction

In humans, the rapid detection and interpretation of facial expressions (FEs) plays a fundamental role in both survival and social functions [1,2]. Converging evidence from neuroimaging and neuropsychological research indicates that FE recognition depends on the integrated engagement of distributed cortical and subcortical networks. More specifically, it has been reported that FE recognition can occur through either a rapid subcortical pathway or a cortical pathway, involving both perceptual and motor processes that are activated within 300 ms following the presentation of static visual stimuli [3]. Regarding the latter and the perceptual pathway activated by them, the initial stages of visual analysis, which may already allow faces to be distinguished from other object categories, recruit areas along the midline occipital cortex approximately 100 ms after stimulus onset. Subsequent processing involves the occipitotemporal cortex, particularly the fusiform gyrus, around 170 ms, as measured through electroencephalography (EEG), more specifically event-related potentials (ERPs) [4,5]. Activity in frontal regions emerging after approximately 200 ms is thought to support the cognitive differentiation of specific emotions and appears to be modulated by task demands [3,6]. Regarding the motor pathway, the processing of FEs also recruits the motor system and their associated cortical networks, which are considered to be directly involved in emotion recognition [4,7,8]. Consistent with this view, several human studies have shown that even during passive viewing of FEs, once visual information reaches limbic structures, such as the cingulate cortex, prefrontal cortex, and amygdala [4,5,9], it engages premotor and motor regions, as well as brainstem nuclei implicated in arousal regulation and emotion-related muscular responses [4]. These findings are supported by neurophysiological investigations employing transcranial magnetic stimulation (TMS), which reported enhanced corticospinal excitability in response to emotional stimuli [10,11,12,13,14].

On the other hand, in the subcortical pathway visual inputs are relayed to the superior colliculus and the pulvinar nucleus of the thalamus, which subsequently project to subcortical structures, including the amygdala, basal ganglia, and brainstem. These regions are thought to mediate rapid, automatic, and coarse-grained processing of emotionally salient stimuli [4,5]. Signals conveyed through this fast subcortical pathway can in turn modulate occipitotemporal and frontal cortical activity via bottom-up mechanisms [5,15,16].

More recently, the cerebellum has been proposed as an additional contributor to FE processing [17,18,19,20,21,22,23,24,25]. Evidence from clinical and neuromodulation studies indicates that both cerebellar lesions [17,18] and alterations in cerebellar plasticity [23,25] impair FE recognition, particularly for negative emotions [17,18,22,23,24,25]. Despite these findings, the pathways by which the cerebellum modulates FE recognition remain incompletely understood.

Anatomically, the cerebellum can influence cortical processing through multiple pathways. It projects to motor and premotor cortices via thalamic relays [26] and, through brainstem connections, interacts with the amygdala [27], which in turn is closely linked to occipitotemporal regions involved in visual and emotional processing [4,5]. However, it is still unclear whether cerebellar contributions to FE recognition rely preferentially on one of these pathways or reflect the combined action of both. Transcranial direct current stimulation (tDCs) is a non-invasive neuromodulator tool which uses electrical current to modulate neural excitability [28]. This technique, when applied to the cerebellar cortex, is able to affect its excitability and plasticity [29,30], modulating both physiological [31,32,33] and behavioral responses [23,33].

In light of the above background, the present study aims to identify the pathway through which the cerebellum contributes to FE recognition processing, assessing how cerebellar tDCs (ctDCs) affects: (a) the motor pathway, involving cerebellar projections to the thalamus and primary motor cortex (M1), probed by TMS protocols; (b) the subjects’ recognition performance, assessed through behavioral tasks; and (c) the perceptual pathway, reflected in the modulation of occipitotemporal cortical excitability, assessed using ERP recordings.

## 2. Methods

### 2.1. Participants

Experiments were conducted in sixteen young healthy volunteers (7 females; mean age 25.0 ± 4.1 years), all right-handed according to the Oldfield Inventory Scale [34]. Sample size was defined based on a priori analysis, with medium Cohen’s f = 0.25, power = 0.8, and α = 0.05, evidencing a minimum sample size of 13 subjects. All subjects gave their informed written consent to participate in the study, which was approved by the local ethical committee (Sardinia Ethics Committee PROT. PROT.-PG/2021/5435) and conducted in accordance with the Declaration of Helsinki. None of the subjects had history or current signs/symptoms of neurological diseases and none was under medications affecting the central nervous system or contraindications to TMS administration [35]. Subjects sat in a comfortable chair and were asked to stay relaxed but alert during the experiments.

### 2.2. Experimental Design

**Figure 1:**
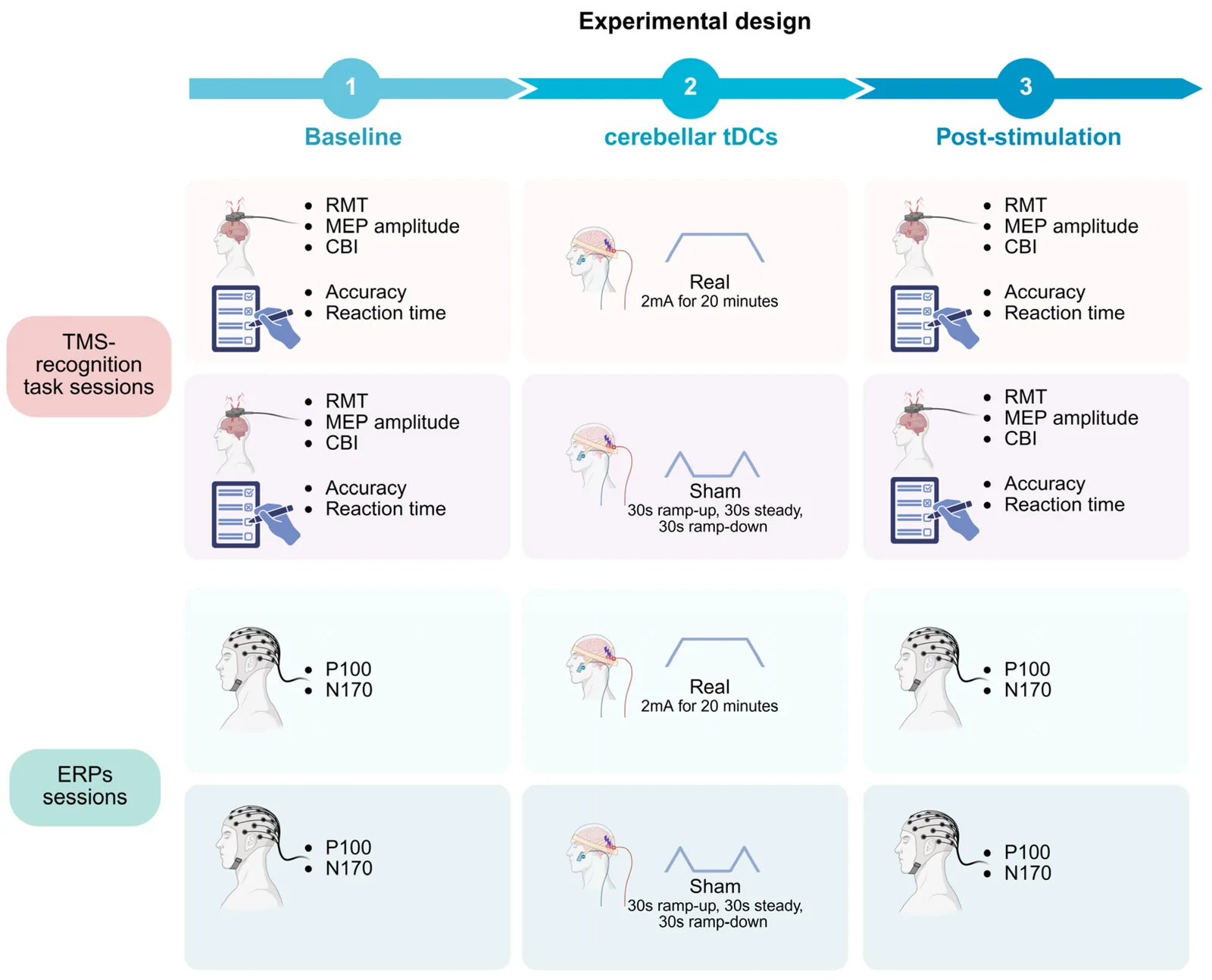
Design of the study. The figure illustrates the overall study design. Participants completed four experimental sessions: two comprehending transcranial magnetic stimulation (TMS) together with recognition task sessions and two event-related potential (ERP) sessions, each performed under either real (cathodal) or sham cerebellar transcranial direct current stimulation (tDCs). The order of the four experimental sessions was randomized, and stimulation conditions were counterbalanced across participants. Sessions were separated by a washout interval of at least one week. During the TMS-recognition task sessions, resting motor threshold (RMT), motor-evoked potentials (MEPs) and cerebellar brain inhibition (CBI) were assessed. In addition, participants performed a facial emotion recognition task, in which accuracy and reaction time were recorded as outcome measures. During the ERP sessions, early visual processing was evaluated by recording the P100 and N170 components. All assessments were performed both before (baseline) and after tDCs administration (post-stimulation).

In all experiments, subjects were required to passively view the FEs, that were presented to them through a computer screen, as detailed below. Every participant completed four experimental sessions performed in randomized order: (1) TMS-recognition task session with real ctDCs; (2) TMS-recognition task session with sham ctDCs; (3) ERP session with real ctDCs; and (4) ERP session with sham ctDCs. Each session was composed of a baseline assessment, followed by tDCs (real or sham), and finally by a post-tDCs evaluation (Figure 1). The sequence of the four experimental sessions was randomized, and stimulation conditions were counterbalanced across participants. To minimize potential carry-over effects of ctDCS and practice-related learning effects, each session was conducted on a separate day, with a washout interval of at least one week between consecutive sessions.

### 2.3. Facial Expression (FE) Stimuli 

Images presented to participants were taken from the Karolinska Directed Emotional Faces (KDEF) database [36]. For our stimuli set, the frontal images of the expressions “neutral”, “happy”, and “fearful” of 8 female and 8 male subjects were selected. Each selected image was replicated twice, and the 96 images were randomly arranged in a sequence, which made up our stimuli set. Visual stimuli for TMS and EEG recordings were shown on a 17” LCD display, with a 1280 × 1024 resolution and a 70 Hz refresh rate, by using the PsychToolbox software (version 3.0.14) [37], running in MATLAB (Version 2021b, MathWorks, Inc., Natick, MA, USA). For all the experimental sessions the duration of the visual stimulus was set at 300 ms. This choice was based on previous studies, which reported 300 ms as an adequate time window for the stimulus to reach M1 and to elicit a bottom-up mechanism able to active occipitotemporal regions [3,14,38].

### 2.4. Transcranial Magnetic Stimulation (TMS)

Cerebellar brain inhibition (CBI) was evaluated using a paired-pulse, dual-coil TMS paradigm. A conditioning stimulus (CS) was delivered over the right cerebellar hemisphere, followed by a test stimulus (TS) applied to the left M1 with an interstimulus interval of 5 ms [39] Stimulation of M1 was performed using a figure-of-eight coil connected to a Magstim 200 stimulator (Magstim Co., Whitland, Dyfed, UK). The optimal scalp position (“hotspot”) for eliciting motor-evoked potentials (MEPs) from the right first dorsal interosseous (FDI) muscle was identified and marked to ensure consistent coil placement throughout the experiment. The coil was oriented tangentially to the scalp at an angle of 45° relative to the interhemispheric midline [40]. The RMT was taken, at the beginning of each session, as the lowest TMS intensity that elicited MEP of 0.05 mV in at least 5 out of 10 consecutive trials and was expressed in percentage of the maximum stimulator output [41]. TS intensity was set at 120% of RMT. The CS was delivered through a double-cone coil positioned 3 cm lateral to the inion, contralateral to M1, at 60% of maximum stimulator output (MSO), in order to avoid activation of the corticospinal tract and brainstem structures [40,41]. MEPs were recorded from the left FDI using a belly–tendon electromyographic montage [40,41]. Signals were amplified (×1000), band-pass filtered (3–3000 Hz), and digitized at 5 kHz using a D360 amplifier (Digitimer Ltd., Welwyn Garden City, UK) and a Power 1401 A/D converter (Cambridge Electronic Design, Cambridge, UK) controlled by Signal 6 software. CBI was quantified as the ratio between conditioned and unconditioned MEP amplitudes. Both baseline and post-tDCs assessments consisted of 16 conditioned and 16 unconditioned MEPs collected for each FE type, resulting in a total of 96 trials.

### 2.5. Event-Related Potentials (ERPs)

Early stages of facial perception were investigated by means of ERP analysis. In particular, two ERP components classically associated with facial processing were examined: (i) the P100 component, a positive deflection peaking approximately 100 ms after stimulus onset, and (ii) the N170 component, a negative deflection emerging around 170 ms following face presentation. Continuous EEG data were acquired using a DC amplifier system (BrainAmp, Brain Products GmbH, Munich, Germany) from 64 scalp electrodes arranged according to the international 10–20 system. Ag/AgCl pellet electrodes were mounted on an elastic cap. The ground electrode was placed at Fpz, whereas Fcz served as the reference site. Signals were digitized at a 5 kHz rate, with the acquisition sampling frequency set to 1000 Hz during recording.

Offline preprocessing was carried out using the EEGLAB toolbox implemented in the MATLAB environment (MathWorks Inc., Natick, MA, USA). Continuous EEG data were segmented into epochs extending from 1 s before to 1 s after stimulus onset. The signal was band-pass filtered between 1 and 100 Hz using zero-phase Butterworth filters, and a 50 Hz notch filter was applied to attenuate line noise.

Artifact-contaminated epochs were initially identified by visual inspection and discarded when gross muscle activity, eye movements, or other non-physiological artifacts obscured the EEG signal. This preliminary rejection involved less than 10% of the epochs. Independent component analysis (INFOMAX-ICA) was subsequently performed, and artifact-related independent components were automatically identified using the TESA component classification procedure. The automatically classified components were then visually inspected, and only those confirmed as representing eye blinks, eye movements, or muscle artifacts were removed before reconstructing the EEG signal. As a final preprocessing step, the data were re-referenced to the common average of all electrodes.

For each participant and experimental condition, P100 and N170 components were quantified in terms of peak amplitude. Analyses were conducted on specific electrode sites: O1, O2, and Oz for the P100 component; P8 and PO8 for the right N170; and P7 and PO7 for the left N170. The P100 amplitude was defined as the maximum positive peak within an 80–110 ms time window across the selected occipital electrodes [42]. Similarly, the N170 amplitude was identified as the maximum negative peak within the 130–200 ms interval across the corresponding posterior temporal electrodes [42]. ERP waveforms were averaged separately for each facial expression condition prior to statistical analysis at both baseline and post-tDCs phases. To reduce participant fatigue and increase the total number of trials, EEG acquisition was divided into two blocks separated by a 2 min rest interval, resulting in approximately 10 min of recording. Each block included 96 visual stimuli, yielding a total of 192 trials per session, with 64 trials for each emotion.

### 2.6. Recognition Task

A response time task was run by the software PsyToolkyt (version 3.0.14) [43], which presented the different FEs and recorded the subjects’ responses. Subjects were instructed to keep the fixation on the screen, visualize each picture, and discriminate, as quickly and as accurately as possible, whether the target face expressed a neutral, happy or fearful face, through the pressing of one of three possible response buttons located in the keyboard, with their right-hand finger. A total of 96 FEs showing neutral, happy, and fearful expressions were shown. The number of correct responses (%) for each FE and the average time taken for each response were used as variables for the statistical analysis.

### 2.7. Cerebellar Transcranial Direct Current Stimulation (ctDCs)

Real and sham ctDCs were delivered using two saline-soaked 5 × 5 cm sponge electrodes (BrainSTIM, EMS srl, Bologna, Italy), with the cathode placed over the right cerebellar hemisphere (1 cm below and 3 cm lateral to the inion) and the anode over the ipsilateral buccinator muscle [30]. Real stimulation was administered as cathodal stimulation, at 2 mA for 20 min, including 30 s ramp-up and ramp-down phases. Cathodal stimulation was selected because the primary aim of the study was to investigate whether modulating cerebellar excitability would alter the physiological and behavioral correlates of facial emotion processing. In healthy young adults, facial emotion recognition performance is typically close to ceiling, limiting the possibility of detecting further improvements following anodal stimulation. Therefore, cathodal stimulation was considered more appropriate for revealing cerebellar contributions by perturbing cerebellar processing. Sham stimulation (30 s ramp-up, 30 s steady, 30 s ramp-down at the beginning and at the end of the tDCs session) mimicked the initial and final somatosensory sensations of active stimulation without producing lasting neuromodulatory effects [30].

### 2.8. Statistical Analysis

All statistical analyses were conducted using SPSS version 26 (SPSS Inc., New York, NY, USA). Data were analyzed through repeated-measures (RM) analyses of variance (ANOVA), followed by planned post hoc *t*-tests with Bonferroni correction applied to adjust for multiple comparisons. The assumption of sphericity was assessed using Mauchly’s test. When violations of sphericity were detected, the Greenhouse–Geisser correction was applied, and the corresponding Mauchly’s test results are reported in Table S1 of Supplementary Materials. The normality test was performed through Kolmogorov–Smirnov and Shapiro–Wilk tests, if most of the variables concerning a single domain (motor, perceptive and recognition) were found significant (*p* < 0.05); Friedman and Wilcoxon tests for post hoc evaluations were performed. Statistical significance was set at *p* < 0.05, effect size was determined through partial eta squared (η^2^*p*).

#### 2.8.1. TMS-Recognition Task Sessions

**TMS**—A paired Student’s *t*-test was performed to evaluate RMT differences between the two sessions (real vs. sham). A preliminary separate three-way RM-ANOVA was performed to investigate the effect of tDCs on unconditioned MEP amplitude during FE processing, with FE type (neutral, happy, fearful), tDCs condition (real vs. sham), and time (baseline vs. post-tDCs) as within-subject factors. To effectively assess the CBI effect at baseline, a three-way RM-ANOVA with tDCs condition (real vs. sham), ISI (unconditioned vs. conditioned MEPs) and FE type (neutral, happy, fearful) as within-subject factors was performed. If a significant effect of ISI but not of FE was detected, a further three-way RM-ANOVA was then conducted on CBI ratios, to assess the cerebellar influence on FE processing. A three-way RM-ANOVA with tDCs condition (real vs. sham), time (baseline vs. post-tDCs) and FE type (neutral, happy, fearful) as within-subject factors was performed.

#### 2.8.2. Recognition Task

Reaction time was separately assessed though a three-way RM-ANOVA with FE type (neutral, happy, fearful), tDCs condition (real vs. sham), and time (baseline vs. post-tDCs) as within-subject factors. On the other hand, accuracy was assessed through the Friedman test for the main effect and the Wilcoxon test for post hoc evaluation, since normality tests were found significant for most of the variables.

#### 2.8.3. ERP Sessions

For the P100 component, a three-way RM-ANOVA was carried out for the wave amplitude, with FE type (neutral, happy, fearful), tDCs (real vs. sham), and time (baseline vs. post-tDCs) as within-subject factors.

For the N170 component, a four-way RM-ANOVA was performed, analyzing the amplitude, with hemisphere (left vs. right), FE type (neutral, happy, fearful), tDCs (real vs. sham), and time (baseline vs. post-tDCs) as within-subject factors.

## 3. Results

### 3.1. TMS-Recognition Task

Table 1 shows RMT and unconditioned MEP mean values throughout the experimental sessions.

Statistical analysis failed to detect any significant effects between the two sessions for RMT, unconditioned MEPs, and CBI. In particular, a paired Student’s *t*-test showed that RMT did not significantly differ between sessions (t_15_ = 0.903; *p* = 0.381; CI95%: −1.28–3.15). The analysis on unconditioned MEPs during passive viewing of FEs showed no significant main effects of FE type (F_2,30_ = 0.562; *p* = 0.576; η^2^*p* = 0.036; Neutral CI95%: 1.25–2.03; Happy CI95%: 1.24–1.99; Fear CI95%: 1.18–1.99), tDCs condition (F_1,15_ = 0.006; *p* = 0.940; η^2^*p* < 0.001; real ctDCs CI95%: 1.05–2.16; sham ctDCs CI95%: 1.16–1.10), or time (F_1,15_ = 0.037; *p* = 0.851; η^2^*p* = 0.002; baseline CI95%: 1.15–2.05; post-ctDCs CI95%: 1.26–1.99) and no significant interactions among factors (*p* > 0.050).

The three-way RM-ANOVA on conditioned and unconditioned MEP amplitude at baseline showed a significant effect of ISI (F_1,15_ = 23.784; *p* < 0.001; η^2^*p* = 0.613; TS CI95%: 1.15–2.05; CS CI95%: 0.86–1.73), showing a reduced MEP amplitude following the CS, but non-significant effects of tDCs condition (F_1,15_ = 0.001; *p* = 0.977; η^2^*p* < 0.001; real ctDCs CI95%: 0.93–1.96; sham ctDCs CI95%: 0.89–2.02), FE type (F_2,30_ = 1.402; *p* = 0.262; η^2^*p* = 0.085; Neutral CI95%: 1.03–1.91; Happy CI95%: 1.04–1.89; Fear CI95%: 0.97–1.87) and interaction among factors (*p* > 0.05).

**Figure 2:**
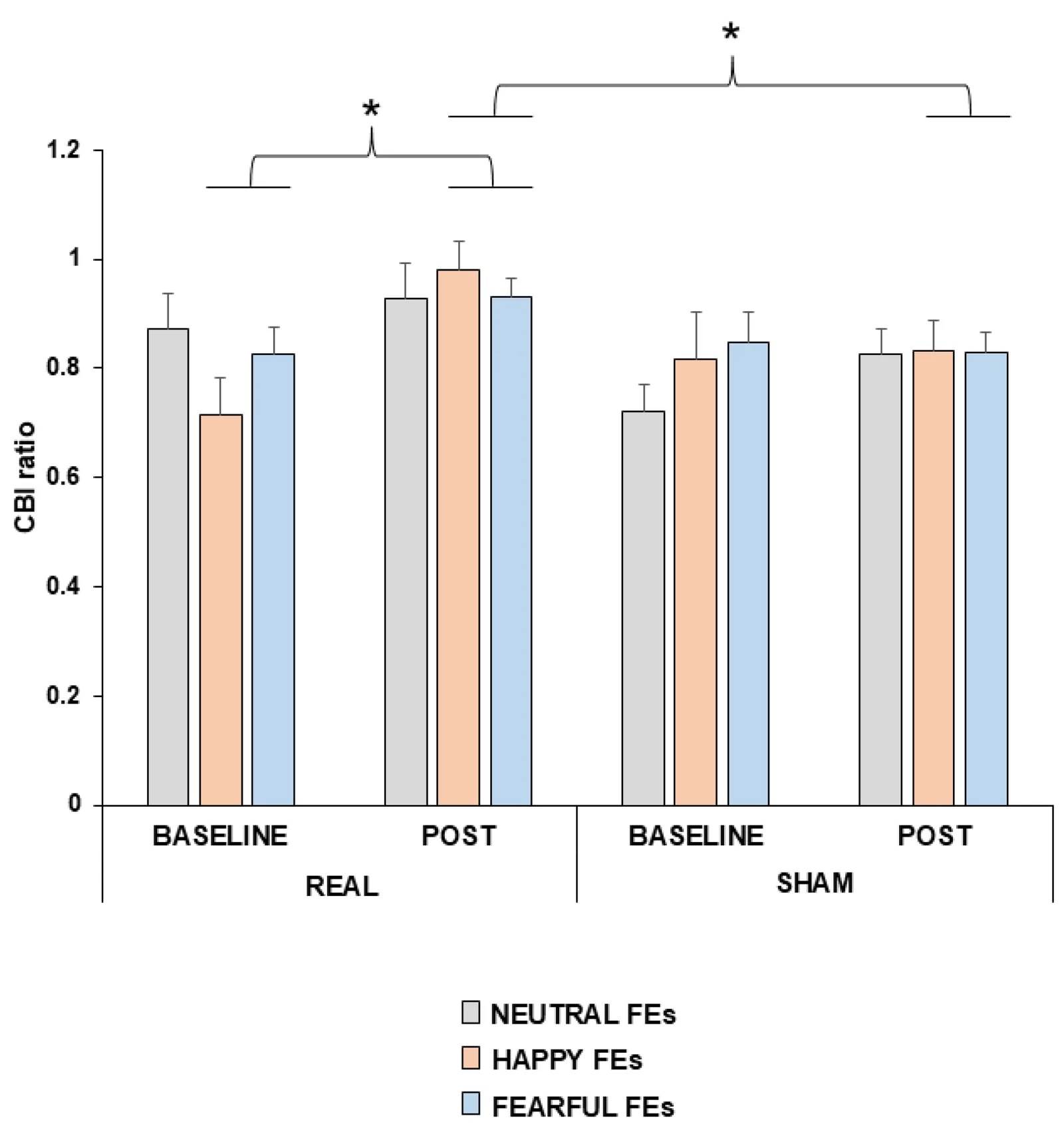
CBI ratio differences between real and sham ctDCs, during the passive viewing of neutral, happy or fearful facial expressions. The graph shows the differences in CBI, assessed as ratio between conditioned and unconditioned motor-evoked potentials (MEPs), between real and sham tDCs, when the subjects passively viewed neutral, happy or fearful FEs. Error bars are represented as standard error of the mean (SEM). * *p* < 0.05.

The analysis of the tDCs effect on the CBI ratio during passive viewing of FEs revealed a trend main effect of tDCs (F_1,15_ = 4.252; *p* = 0.057; η^2^*p* = 0.221; real ctDCs CI95%: 0.79–0.96; sham ctDCs CI95%: 0.73–0.89), with higher CBI ratio following real compared to sham tDCs and a trend of tDCs × time interaction (F_1,15_ = 4.123; *p* = 0.060; η^2^*p* = 0.216), whereas the effect of FE type was not significant (F_2,30_ = 0.327; *p* = 0.723; η^2^*p* = 0.021; Neutral CI95%: 0.74–0.93; Happy CI95%: 0.73–0.94; Fear CI95%: 0.80–0.91). In contrast, a significant main effect of time emerged, with higher post-tDCs CBI ratios compared to pre-tDCs (F_1,15_ = 11.790; *p* = 0.004; η^2^*p* = 0.440; baseline CI95%: 0.71–0.89; post-ctDCs CI95%: 0.81–0.97). Additionally, a significant three-way interaction among FE type × tDCs × time was found (F_2,30_ = 3.472; *p* = 0.044; η^2^*p* = 0.188). Post hoc analysis revealed a significantly higher CBI ratio after real tDCs compared to pre-real tDCs during the viewing of happy FEs (*p* = 0.001; CI95%: −0.41–−0.13), as well as a trend toward significance for fearful FEs (*p* = 0.058; CI95%: −0.22–−0.004), but not for neutral FEs (*p* = 0.296, CI95%: −0.16–0.05). Furthermore, in the post-tDCs assessment, CBI ratio was significantly higher following real than sham tDCs for happy (*p* = 0.037, CI95%: 0.01–0.29) and fearful FEs (*p* = 0.044, CI95%: 0.003–0.21), but not for neutral FEs (*p* = 0.111, CI95%: −0.03–0.23) (Figure 2).

### 3.2. Recognition Task

**Figure 3:**
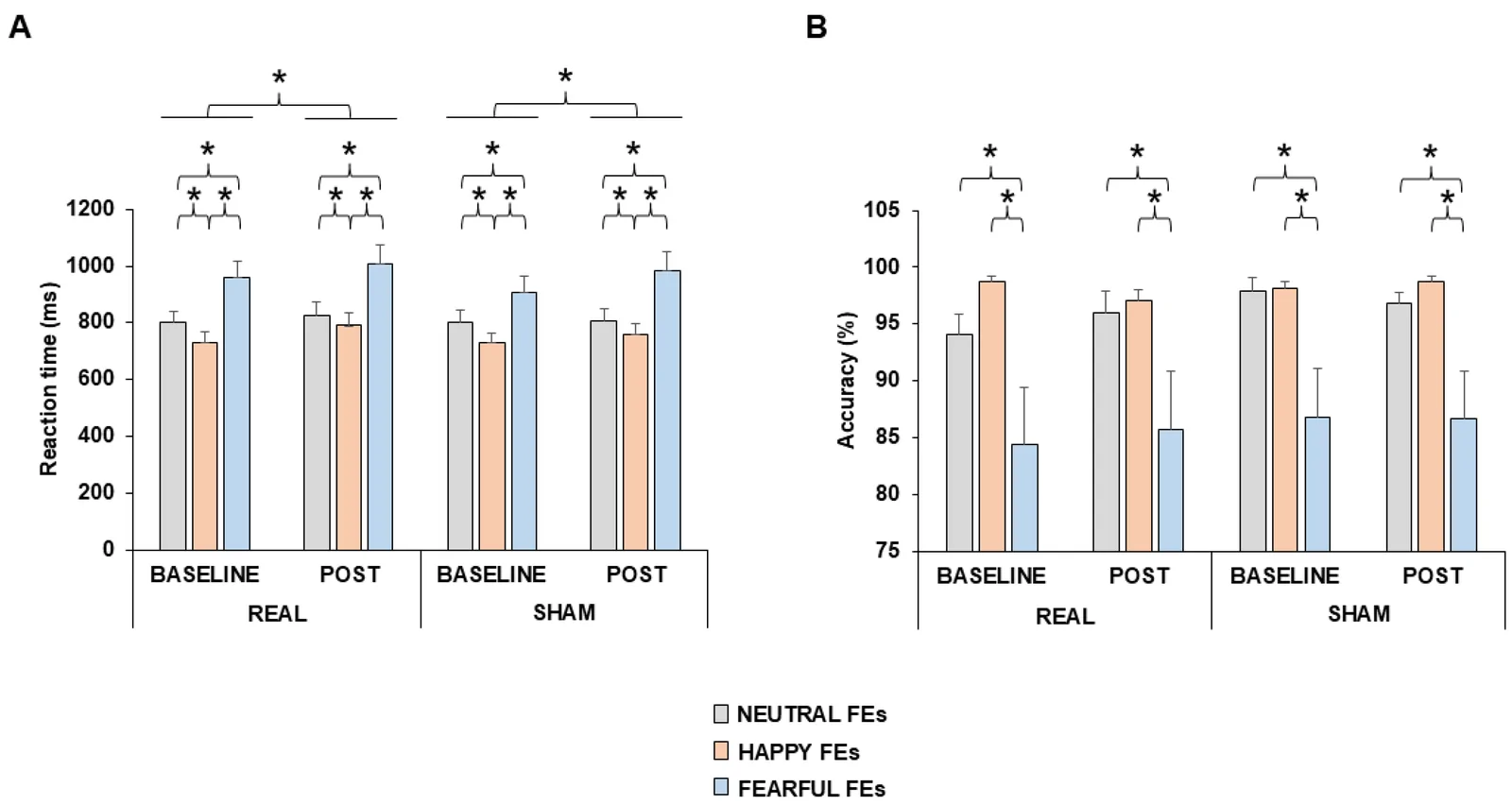
Reaction times and accuracy differences between real and sham ctDCs, when subjects passively viewed neutral, happy or fearful facial expressions. The bar charts show the differences in reaction time (**A**) and accuracy (%) (**B**) in the recognition task, between real and sham ctDCs, when the subjects passively viewed neutral, happy or fearful facial expressions (FEs). Error bars are represented as standard error of the mean (SEM). * *p* < 0.05.

The analysis of reaction times revealed significant main effects of FE type (F_2,30_ = 31.752; *p* < 0.001; η^2^*p* = 0.678; Neutral CI95%: 723.70–894.03; Happy CI95%: 674.11–829.93; Fear CI95%: 848.79–1081.71) and time (F_1,15_ = 7.777; *p* = 0.014; η^2^*p* = 0.341; baseline CI95%: 738.22–904.48; post-ctDCs CI95%: 766.96–958.53), with longer post-tDCs reaction times compared to baseline, but no significant effect of tDCs (F_1,15_ = 0.779; *p* = 0.391; η^2^*p* = 0.049, real ctDCs CI95%: 758.62–946.17; sham ctDCs CI95%: 742.02–921.37) or interactions among factors (*p* > 0.050) (Figure 3A). Post hoc analysis of FE condition, indicated regardless that participants responded more slowly to fearful FEs than to happy (*p* < 0.001; CI95%: 129.34–297.12) and neutral FEs (*p* = 0.002, CI95%: 60.67–252.10), and more slowly to neutral than to happy FEs (*p* < 0.001, CI95%: 32.06–82.63).

The Friedman analysis concerning accuracy showed a significant main effect (χ^2^_16_ = 57.780; *p* < 0.001). Post hoc analysis conducted through Wilcoxon test resulted in significance only when comparing FEs, regardless of tDCs and time. In particular, accuracy was higher when subjects viewed neutral compared to fearful FEs (z = −3.995; *p* < 0.001) as well as when viewing happy FEs rather than fearful ones (z = −5.618; *p* < 0.001) (Figure 3B).

### 3.3. ERP

**Figure 4:**
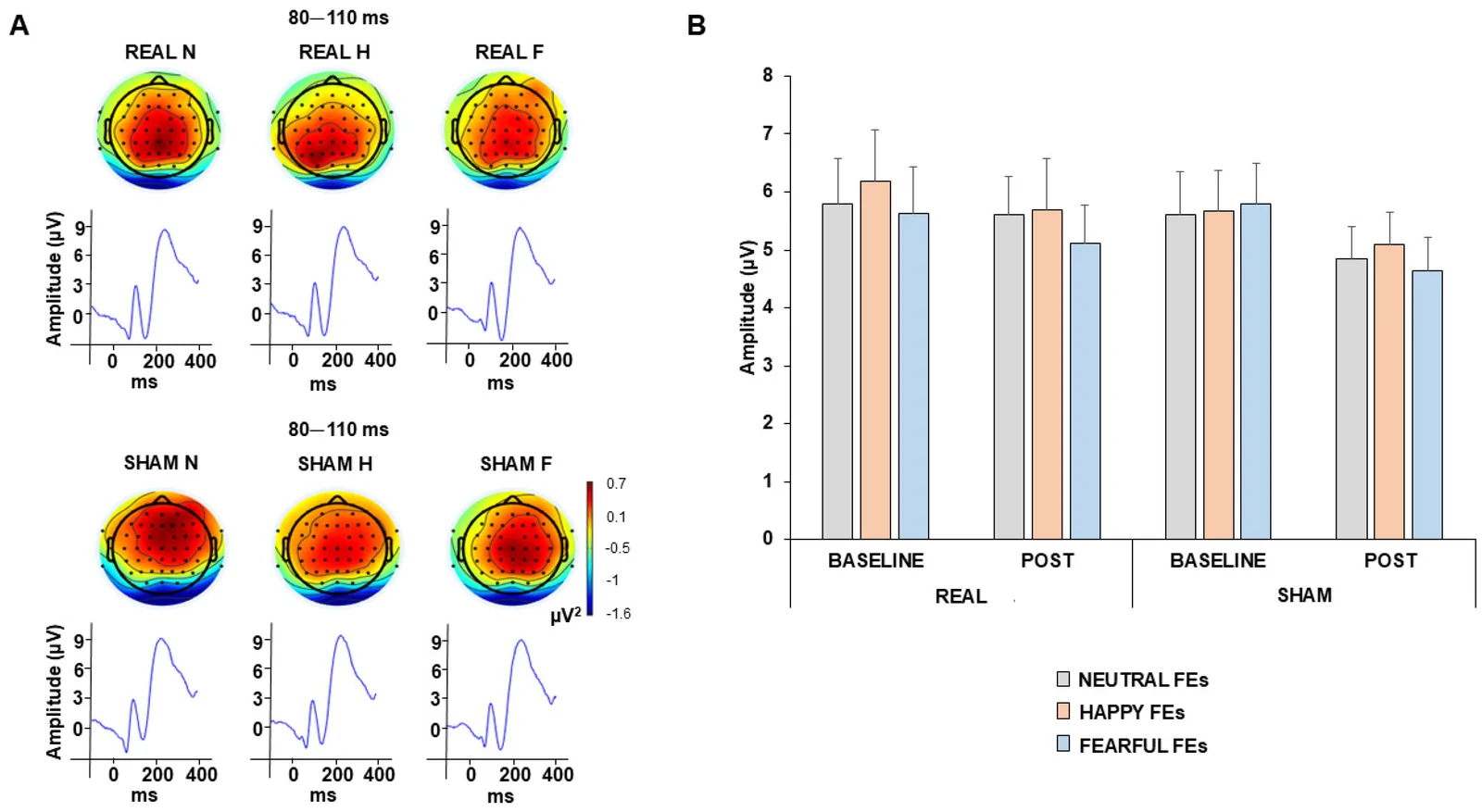
P100 wave differences between real and sham ctDCs, when subjects passively viewed neutral, happy or fearful facial expressions. Panel (**A**) shows the P100 topography and waveform averaged across the baseline and post-ctDCs time points, for the real and sham stimulation sessions, while participants passively viewed neutral (N), happy (H), or fearful (F) facial expressions (FEs). The bar chart in panel (**B**) shows the differences in the P100 wave amplitude between real and sham ctDCs, when the subjects passively viewed neutral, happy or fearful FEs. Error bars are represented as standard error of the mean (SEM).

Statistical analysis of the P100 amplitude revealed no significant effects of FE type (F2,30 = 2.403; p = 0.109; η2p = 0.146; Neutral CI95%: 4.16–6.78; Happy CI95%: 4.17–7.15; Fear CI95%: 3.99–6.59), tDCs (F1,15 = 0.714; p = 0.412; η2p = 0.049, real ctDCs CI95%: 4.06–7.29; sham ctDCs CI95%: 4.02–6.53), time (F1,15 = 3.558; p = 0.080; η2p = 0.203, baseline CI95%: 4.23–7.32; post-ctDCs CI95%: 3.93–6.40) or interactions among factors (*p* > 0.050) (Figure 4).

**Figure 5:**
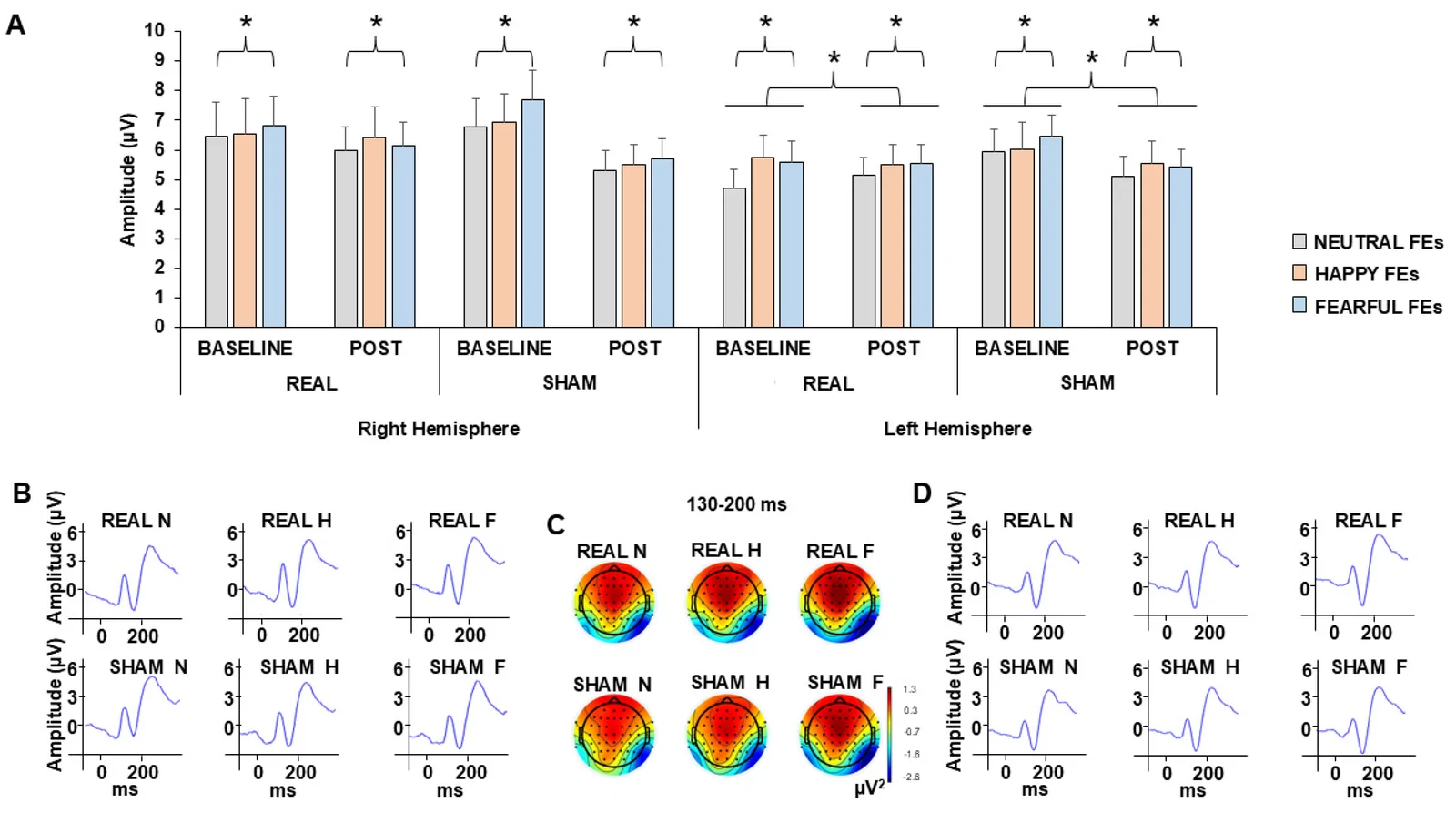
N170 wave differences between real and sham ctDCs, when subjects passively viewed neutral, happy or fearful facial expressions. The bar chart shows the differences in the N170 amplitude between real and sham ctDCs, separately for the left and right hemispheres, during passive viewing of neutral, happy, and fearful facial expressions (FEs) (**A**). Error bars are represented as standard error of the mean (SEM). * *p* < 0.05. Panels (**B**,**D**) show the N170 waveforms averaged across the baseline and post-tDCs time points for the left and right hemispheres, respectively, under the real and sham stimulation sessions. Panel (**C**) shows the corresponding N170 topography averaged across the baseline and post-tDCs time points for the real and sham stimulation sessions.

A four-way RM-ANOVA on the N170 amplitude revealed no significant main effects of hemisphere (F_1,15_ = 1.271; *p* = 0.279; η^2^*p* = 0.083, right CI95%: 8.04–4.64; left CI95%: 6.77–4.26), tDCs (F_1,15_ = 0.185; *p* = 0.674; η^2^*p* = 0.013; real ctDCs CI95%: 7.18–4.47; sham ctDCs CI95%: 7.42–4.64), or time (F_1,15_ = 4.099; *p* = 0.062; η^2^*p* = 0.226; baseline CI95%: 7.73–4.79; post-ctDCs CI95%: 6.75–4.44). However, significant main effects of FE type (F_2,30_ = 3.700; *p* = 0.038; η^2^*p* = 0.209, Neutral CI95%: 6.89–4.36; Happy CI95%: 7.42–4.57; Fear CI95%: 7.35–4.97) and interaction hemisphere × time (F_1,15_ = 9.429; *p* = 0.008; η^2^*p* = 0.402) were found. Post hoc analyses indicated higher N170 amplitudes for fearful compared to neutral FEs (*p* = 0.011, CI95%: −0.96–0.12). The hemisphere × time interaction showed that the N170 amplitude decreased from baseline to post-tDCs only in the right hemisphere (*p* = 0.022; CI95%: −1.83–0.17) (Figure 5).

## 4. Discussion

The results showed that ctDCs reduced the CBI of the hand area of M1, particularly when participants passively viewed emotional FEs. However, ctDCs did not affect the perceptual processing of FEs and recognition performance. Taken together, these findings suggest that the cerebellum’s contribution to FE processing is mediated more strongly through its connections with motor regions than with perceptual ones.

ctDCs was found to modulate the cerebello–thalamo–M1 pathway (as assessed through CBI), with stronger effects observed during the viewing of emotional FEs. To date, only one neurophysiological study has shown that modulation of activity in the posterior cerebellar hemispheres influences M1 excitability during the observation of emotional FEs [22]. In particular, using repetitive TMS, the authors reported enhanced M1 activity when participants viewed negative FEs. However, in the study by Ferrari and colleagues [22], CBI was not assessed, as the authors focused exclusively on MEPs recorded from the hand representation area of M1. Importantly, unconditioned MEPs may be influenced not only by cerebellar activity but also by the activation of multiple cortical and subcortical pathways, including those originating from the cerebellum [44]. Therefore, the observed effects on unconditioned MEPs did not clarify which specific pathway was involved in FE processing. In contrast, in the present study, unconditioned MEPs were not affected by ctDCs, possibly due to differences in the experimental design, such as the neuromodulation protocol (repetitive TMS vs. tDCs), the cerebellar hemisphere targeted (left vs. right hemisphere), or the FEs presented (neutral and fearful vs. happy, neutral, and fearful expressions). Alongside the non-significant effect on unconditioned MEPs, we found a robust effect on CBI, suggesting a modulation of the cerebello-M1 pathway during the processing of happy and fearful FEs.

The neural circuitry underlying CBI has been extensively characterized [44,45,46]. In particular, it has been demonstrated that applying TMS over the cerebellum leads to the activation of Purkinje cells (PCs), thereby increasing their inhibitory output to the dentate nucleus (DN). This, in turn, reduces the excitatory drive from the DN to M1 via thalamic projections, resulting in the suppression of motor output and a reduction in the conditioned MEP amplitude relative to the unconditioned MEP elicited by a single pulse over M1 [39]. Previous studies have shown that ctDCs induces LTD-like plasticity mechanisms, reducing the inhibitory influence of PCs on the DN. This, in turn, leads to the disinhibition of motor cortical activity, ultimately resulting in a reduction in CBI [44]. In parallel, the lateral part of the cerebellar cortex not only serves as a motor control pathway, but several studies have also shown that lobules VI, VII, VIII, and Crus I–II of the lateral posterior cerebellar cortex are involved in cognitive and emotional processing [26,47,48]. These regions receive inputs from the prefrontal, posterior parietal, superior temporal, parastriate, parahippocampal, and cingulate cortices [48,49,50,51], all of which contribute to the processing of cognitive and affective information. Although cerebellar function is organized into largely closed-loop circuits [26], whereby individual cerebellar modules project back to the same cortical regions from which they receive input, evidence suggests that communication between different loops also occurs, enabling the integration and transfer of information across functional domains [26]. Accordingly, emotional information conveyed by FEs and processed within lateral posterior cerebellar regions, particularly lobules VI, VII, VIII, and Crus I–II, may, through the DN, influence not only the prefrontal, temporal, and cingulate networks but also the M1. Such interactions may provide a neural mechanism through which emotional FEs influence cerebello–motor network activity. One possible interpretation is that this modulation may contribute to motor simulation processes. Specifically, premotor information related to simulation mechanisms is conveyed to the cerebellum, where it is integrated with internal and external information to fine-tune motor preparation processes. This mechanism may refine preparatory motor responses, thereby modulating CBI selectively during the passive viewing of emotional, rather than neutral FEs as suggested by our CBI findings. However, alternative explanations, such as increased emotional arousal, enhanced attentional engagement, or general motor readiness elicited by emotionally salient stimuli, cannot be excluded, as these mechanisms were not directly assessed in the present study.

The CBI results showed that cerebellar physiology became sensitive to emotional facial cues (happy and fearful faces) only following tDCs, whereas no emotion-related modulation was observed under baseline conditions. This finding is consistent with previous studies reporting effects of emotional FEs on cerebellar physiology only after plasticity protocols applied over the cerebellum [22,24]. Taken together, these observations may be interpreted according to two hypotheses. On the one hand, under normal cerebellar functioning, emotional FE processing may rely predominantly on other cortical and subcortical networks, whereas cerebellar contributions become more evident following plastic changes (either in PCs or DN connectivity), leading to a greater involvement of cerebellar circuits in FE processing. This interpretation is further supported by the emotion recognition results. Indeed, differences in recognition accuracy across emotions were already present at baseline, in agreement with previous findings [23,25]. On the other hand, it is possible that, in healthy subjects, a ceiling effect characterizes the neural pathways involved in FE processing, limiting the possibility of observing further enhancement under normal conditions. In this context, neuromodulation may be necessary to perturb or reshape the recruited pathways, thereby enabling a more precise investigation of their contribution to FE processing.

No statistically significant differences for ctDCs were observed on the perceptual stage of FE processing, and the confidence intervals indicate that the data are compatible with a range of effects, including small effects. Therefore, these findings should be interpreted as an absence of detectable effects under the present experimental conditions rather than evidence of no effect. Indeed, the effect sizes associated with the stimulation-related effects on the P100 and N170 amplitudes were small, suggesting that any modulation of early occipitotemporal processing, if present, was limited under the present experimental conditions. However, given the sample size, subtle effects cannot be completely excluded. Occipitotemporal regions play a crucial role in the early perception and discrimination of FEs [4,5], although conscious recognition is thought to emerge only after approximately 300 ms from the visual stimuli, reflecting the time required for information to reach frontal cortical areas [3]. In line with the present findings, a previous study in patients with cerebellar ischemic lesions found no significant alterations in occipitotemporal processing specifically related to facial emotion discrimination [18]. Conversely, the cerebellum may play a role in the early perceptual stages of face identity processing, without a clear contribution to emotional processing. This interpretation is supported by recent evidence showing altered N170 amplitudes in patients with cerebellar infarction during a face discrimination task involving scrambled facial stimuli [52]. Thus, it is possible to hypothesize that the cerebellum influences perceptual processing within occipitotemporal networks involved in face perception, although this contribution may not directly involve emotional processing. Rather, its role in emotional FE processing may emerge at later processing stages through interactions with higher-order cognitive and motor networks, including attentional allocation, higher-order perceptual integration, decision-making, response selection, or emotional evaluation. These later processes are typically associated with ERP components occurring after N170 that were not investigated in the present study.

Finally, in the present study, ctDCs did not modulate FE recognition performance, apparently in disagreement with a previous finding reporting opposing results [23]. One possibility for this result is that the emotion recognition task may have lacked sufficient sensitivity to detect any modulation by tDCs. In particular, the relatively small number of facial stimuli, selected to limit the overall duration of the experiment, may have reduced the statistical power to detect such effects, which may represent a potential limitation for the present study.

Taken together, these findings suggest that the cerebellum does not play a primary role in FE discrimination. Nevertheless, cerebellar dysfunction appears capable of impairing emotion recognition abilities, as consistently reported in patients with cerebellar damage [17,18,20]. Thus, rather than serving as a core structure for facial emotion recognition, the cerebellum may contribute to optimizing and refining the processing of emotional facial information, probably through learning mechanisms which modulate cerebellar plasticity.

Some limitations should be acknowledged when interpreting the present findings. First, although the sample size was determined by an *a priori* power analysis and was sufficient to detect medium-sized effects, it remains relatively small and may have limited the detection of subtle neuromodulatory effects, particularly for ERP and behavioral outcomes. Second, only three FE types (neutral, happy, and fearful) were investigated using a relatively limited number of stimuli. Including a broader range of emotional expressions and a larger stimulus set may improve the sensitivity of future studies. Third, ERP analyses were restricted to the early P100 and N170 components, which primarily reflect the initial visual-perceptual stages of face processing. Therefore, the present results do not exclude possible cerebellar contributions to later stages of emotional face processing, which could be explored by analyzing later ERP components (e.g., P2, N2, P3, or late positive potentials). Finally, only cathodal ctDCs was examined. Future studies comparing cathodal and anodal stimulation, as well as different stimulation protocols, may provide a more comprehensive understanding of cerebellar contributions to emotional face processing.

## 5. Conclusions

In conclusion, the present study suggests that ctDCs modulates cerebellar involvement in the processing of happy and fearful FEs. This action is exerted mainly through reduced inhibition of the cerebellar–thalamo–M1 pathway, as assessed by CBI. In contrast, early perceptual mechanisms and FE recognition performance were not affected. Overall, these findings indicate that the cerebellum contributes to FE processing primarily through modulatory influences on motor networks rather than through early perceptual mechanisms.

## Figures and Tables

**Table 1:** RMT and unconditioned MEP mean values.

		Cathodal ctDCS		Sham ctDCS	
RMT (% MSO)		40.75 ± 8.36		39.81 ± 7.47	
		**Baseline**	**Post-tDCS**	**Baseline**	**Post-tDCS**
Unconditioned MEP (mV)	N	1.54 ± 1.01	1.70 ± 1.19	1.64 ± 1.08	1.69 ± 0.85
	H	1.67 ± 1.04	1.62 ± 1.07	1.53 ± 1.23	1.64 ± 0.72
	F	1.54 ± 1.11	1.53 ± 1.10	1.69 ± 1.22	1.56 ± 0.79

RMT, resting motor threshold; MSO, maximum stimulator output; ctDCS, cerebellar transcranial direct current stimulation; N, neutral facial expressions; H, happy facial expressions; F, fearful facial expressions; MEP, motor-evoked potential; mV, millivolts. Error measurement, ± standard deviation.

## Data Availability

Dataset available on request from the authors—the raw data supporting the conclusions of this article will be made available by the authors upon reasonable request.

## Supplementary Materials

The following supporting information can be downloaded at: https://www.mdpi.com/article/10.3390/brainsci16080858/s1, Table S1: Mauchly’s sphericity test results.
