# Supplementary material for: Cerebellar Transcranial Direct Current Stimulation Induces a Predominant Motor Rather than Perceptive Contribution to Emotion Facial Expression Processing
Source: Brain Sci. 2026 Aug 13;16(8):858. doi: 10.3390/brainsci16080858 (PMC13511599; doi:10.3390/brainsci16080858)
Supplement: Supplementary file 1 [file brainsci-16-00858-s001.zip › brainsci-4465543-supplementary.pdf]

Supplementary Materials

Table S1. Mauchly's sphericity test results.

Variable analyzed	Mauchly's parameters						
	**Factors and Interaction**	**W**	**X^2**	**DF**	***p*-value**	**Corr**	**GG ε**
TMS parameters							
Unconditioned MEP (TS only)	FE type	0.851	2.261	2	0.323	NO	0.975
	tDCs x FE type	0.779	3.490	2	0.175	NO	0.906
	Time x FE type	0.697	5.060	2	0.080	NO	0.767
	tDCs x time x FE type	0.828	2.643	2	0.267	NO	0.853
Unconditioned MEP (TS vs CS)	FE type	0.749	4.038	2	0.133	NO	0.800
	tDCs x FE type	0.953	0.667	2	0.716	NO	0.956
	ISI x FE type	0.999	0.015	2	0.993	NO	0.999
	tDCs x ISI x FE type	0.933	0.972	2	0.615	NO	0.937
CBI ratio	FE type	0.794	3.234	2	0.199	NO	0.829
	tDCs x FE type	0.985	0.213	2	0.899	NO	0.985
	Time x FE type	0.924	1.103	2	0.576	NO	0.930
	tDCs x time x FE type	0.991	0.125	2	0.939	NO	0.991
Recognition task							
Reaction times	FE type	0.159	25.708	2	<0.001	YES	0.543
	tDCs x FE type	0.952	0.696	2	0.706	NO	0.954
	Time x FE type	0.699	5.010	2	0.082	NO	0.769
	tDCs x time x FE type	0.851	2.255	2	0.324	NO	0.870
ERP							
P100 Amplitude	FE type	0.943	0.761	2	0.684	NO	0.946
	tDCs x FE type	0.842	2.243	2	0.326	NO	0.863

	Time x FE type	0.788	3.103	2	0.212	NO	0.825
	tDCs x time x FE type	0.972	0.365	2	0.833	NO	0.973
N170 Amplitude	FE type	0.686	4.899	2	0.086	NO	0.761
	Hemisphere x FE type	0.601	6.620	2	0.037	YES	0.715
	tDCs x FE type	0.632	5.965	2	0.051	NO	0.731
	Hemisphere x tDCs x FE type	0.590	6.850	2	0.033	YES	0.709
	Time x FE type	0.841	2.255	2	0.324	NO	0.863
	Hemisphere x time x FE type	0.458	10.139	2	0.006	YES	0.649
	tDCs x time x FE type	0.838	2.291	2	0.318	NO	0.861
	Hemisphere x tDCs x time x FE type	0.609	6.446	2	0.040	YES	0.719

W, Mauchly's statistic; X^2, Approximate chi square statistic; DF, degrees of freedom; Corr, correction; GG ε, Greenhouse–Geisser epsilon; TMS, transcranial magnetic stimulation; MEP, motor-evoked potential; CBI, cerebellar brain inhibition; TS, test stimulus; CS, conditioning stimulus; FE face expression; tDCs, transcranial direct current stimulation.
